# Larger Fig Wasps Are More Careful About Which Figs to Enter – With Good Reason

**DOI:** 10.1371/journal.pone.0074117

**Published:** 2013-09-23

**Authors:** Cong Liu, Da-Rong Yang, Stephen G. Compton, Yan-Qiong Peng

**Affiliations:** 1 Key Laboratory of Tropical Forest Ecology, Xishuangbanna Tropical Botanical Garden, Chinese Academy of Sciences, Kunming, China; 2 Okinawa Institute of Science and Technology Graduate University, Okinawa, Japan; 3 Department of Zoology and Entomology, Rhodes University, Grahamstown, South Africa; USDA-Agricultural Research Service, United States of America

## Abstract

Floral longevity reflects a balance between gains in pollinator visitation and the costs of flower maintenance. Because rewards to pollinators change over time, older flowers may be less attractive, reducing the value of extended longevity. Un-pollinated figs, the inflorescences of *Ficus* species, can remain receptive for long periods, but figs that are older when entered by their host-specific fig wasp pollinators produce fewer seeds and fig wasp offspring. Our field experiments with *Ficus* *hispida* , a dioecious fig tree, examined how the length of time that receptive figs have remained un-pollinated influences the behaviour and reproductive success of its short-lived fig wasp pollinator, *Ceratosolensolmsi* *marchali*. The results were consistent in three different seasons, and on male and female trees, although receptivity was greatly extended during colder months. Pollinators took longer to find the ostioles of older figs, and longer to penetrate them. They also became increasingly unwilling to enter figs as they aged, and increasing numbers of the wasps became trapped in the ostiolar bracts. Larger individuals were particularly unwilling to enter older figs, resulting in older figs being pollinated by smaller wasps. On female trees, where figs produce only seeds, seed production declined rapidly with fig age. On male trees, the numbers and size of fig wasp offspring declined, and a higher proportion were male. Older male figs are harder to enter, especially for larger individuals, and offer poorer quality oviposition opportunities. This study opens an interesting new perspective on the coevolution of figs and their pollinators, especially factors influencing pollinator body size and emphasises the subtleties of interactions between mutualists.

## Introduction

Natural selection continuously sculpts body size [1]. In insects, body size is a heritable trait that is nonetheless subject to strong environmental modification [2–4] linked to variables such as the length of time that a larva has fed, ambient temperatures and the quantity and quality of its food [1,5]. Fitness is generally positively correlated with body size in insects [6], with larger males more successful at gaining mates and larger females able to lay more eggs, yet body size stability within species is maintained from generation to generation [7].

Factors maintaining equilibrium body sizes are poorly understood, especially selection pressures operating against larger individuals [8–10]. During one stage of their life cycle, the female fig wasps (Agaonidae) that pollinate the inflorescences (figs) of fig trees (*Ficus*) can provide an unusually clear example of selection acting against larger individuals – they are more likely to become stuck when they try to force their way into the centre of the figs where they lay their eggs [11]. Selection at other stages of their life cycle nonetheless favours larger females because they are more likely to successfully reach trees with host figs [11,12] and larger females contain more eggs, so should have higher fecundity in figs where oviposition sites are not limiting (C. Liu unpublished). This results in contrasting directional selection on body size acting in sequence, with larger, then smaller, and then larger adult females favoured at different times during their short adult life spans.

The partnership between fig trees and fig wasps is characterized by extreme specificity and high levels of behavioural and morphological co-adaptation, much of it linked to the unusual structure of the *Ficus* inflorescence – the fig. Figs (sometimes called syconia) are complex inflorescences with their flowers entirely enclosed around an inner chamber (lumen). The only route to the flowers for pollen and pollinators from the outside is through a narrow slit-like pore called the ostiole. Only highly specialized fig wasps can enter the figs and pollinate them. The pollinators of each *Ficus* species are drawn to their host plants by species- and stage-specific volatile blends [13,14]. The temporary partial opening of the ostiole allows the female pollinators to gain entry to the cavity [15], from where they can lay their eggs into some of the ovules [16]. At this time they also pollinate the flowers [17,18]. One pollinator offspring can develop in each ovule. After a few weeks, the adults mate and emerge from their galls, and the females then become loaded with pollen before escaping through a hole cut through the fig wall by the males. They then fly off in search of figs that are suitable for entry, which are usually on other trees. Adult female longevity is short, rarely exceeding 48 hours [19].

Figs start to become less attractive to other pollinators a few hours after they have been entered, though this is postponed in some species if they have only received a single pollinator [20,21]. The cessation of release of attractant volatiles may reflect both the termination of production and closure of the ostiole. Figs that have not been pollinated can remain receptive for much longer. Floral longevity (the length of time that a flower remains available for pollination) varies greatly among different plant species, and reflects a balance between the benefits of greater likelihood of pollinator visitation and the cost of flower maintenance [22–25]. Figs display extended floral longevity and if they are not entered by fig wasps they remain attractive to pollinators for days or weeks [20,21,26], but prolongation of receptivity may come at the cost of reduced reproductive efficiency among older figs [20]. Two studies have reported that pollinator females are more likely to enter younger figs [21,26], though the proximal causes of this preference have not been established. Fig wasps may be responding to subtle changes in the quality as well as the quality of volatiles released as figs age, because higher numbers of offspring (and seeds) have been recorded from figs that were pollinated shortly after becoming receptive, with productivity then declining progressively with the length of time that the figs had waited to be pollinated [21,26]. These studies also reported more subtle changes in the reproductive success of the pollinators, with sex ratios among offspring becoming less female-biased in older figs. This could reflect the smaller clutches sizes that were achieved in the older figs, with male eggs laid at the start of oviposition sequences [27].

The ostiole acts as a physical filter that prevents non-adapted insects from reaching the centre of the fig, where the flowers are then readily accessible. The difficulties associated with penetration of the ostiole are reflected in numerous morphological adaptations in adult female figs wasps that include a flattened head, mandibular appendages, and short, strong and spiny legs [18]. The ostiole also strongly influences the relative length and width of fig wasp heads [28,29] and sets an upper limit to pollinator head and body size [11,12]. Along with contributing to the specificity of fig wasp entry (and the identity of the pollen they carry), the ostiole strongly constrains the subsequent behaviour of the pollinators because it removes the distal segments of their antennae and strips off their wings when they are passing through [30]. Most pollinators never leave the first fig they enter, and those that do so are unable to disperse to other trees [31,32].

Foundresses can find it more difficult to penetrate the ostioles of older figs [21,32] and larger foundresses can also be more likely to became trapped in the ostioles than smaller females [11]. Together, these suggest that successful passage through the ostiole is most likely among smaller foundresses attempting to enter younger figs that have recently become receptive, with selection for smaller body size also less intense at that time.

In this study we used controlled experiments to examine the interplay between host floral longevity, ostiole permeability and body size in fig wasps. We asked (1) Does the willingness of pollinators to enter figs vary with the length of time that a fig has waited to be pollinated (fig age)? (2) Does ease of passage through the ostiole change with fig age? (3) Does successful entry into the figs vary according to the size of the wasps? (4) Are age-related changes similar in male and female figs? And (5) Do pollinators that enter older figs produce fewer or smaller offspring, with different sex ratios? These questions were examined using crops that were produced during three different seasons, because floral longevity varies according to ambient temperatures [33,34].

## Materials and Methods

### Study site and species

Xishuangbanna Tropical Botanical Garden (XTBG) is located in southwest China (21°55′N, 101°15′E, at about 555 m asl). Annual temperatures (1960-2000) average 21.8°C, with means of 25.7°C in the hottest month (June) and 16.0°C in the coldest month (January) (Xishuangbanna Forest Ecology Station, 2001). There are three well defined seasons, warm and rainy (June to October), cool and dry (November to February) and warm and dry (March to May). No specific permission was required for these locations/activities. XTBG is not privately-owned and is available for research by scientists based in the garden. Field studies did not involve endangered or protected species.

*Ficus* *hispida* L. is a small free-standing functionally dioecious tree widely distributed across SE Asia [35]. Figs on male trees contain male flowers and female flowers that act as nurseries for developing fig wasp larvae, whereas figs on female trees contain only female flowers and produce only seeds. Both sexes produce figs all year round, usually in discrete synchronized crops, on leafless branchlets hanging down from the trunk and larger branches [36]. The tree is pollinated locally by *Ceratosolensolmsi* *marchali* Mayr. Figs on female trees contain 2531.43 ± 50.20 (mean ± SE, *n* = 60) female flowers, and on male trees contain 1774.33 ± 48.79 (*n* = 92) female flowers. Under natural conditions, the figs are entered by between 1 and 31 foundress fig wasps (5.22 ± 5.19 in females and 6.00 ± 6.11 in males), but many fail to penetrate the ostiole and therefore fail to reproduce or pollinate [37]. Foundresses have not been observed to re-emerge from figs after entry. The extent of heritability of body size in *C. smarchali* is unknown, but body size varies according to where in a fig a larva develops, suggesting a strong influence of host quality (Y.-Q. Peng, unpublished).

### Floral longevity in the absence of pollination

We enclosed young pre-receptive figs on one female and one male tree in netting bags to prevent oviposition by pollinators and other fig wasps in October 2010, January 2011 and May 2011, during the warm rainy, cold dry and warm dry seasons respectively. We determined the onset of receptivity on the basis of pollinator behavior: if freshly-emerged pollinators placed on a fig succeeded in partially gaining entry within five minutes (at which point they were removed), the fig was deemed receptive. If still pre-receptive, the bag was replaced and the fig was tested again in the same way the following day. We recorded the duration of the receptive period after the first day on which pollinators were willing to enter at daily intervals by again placing females on the bagged figs and recording whether or not they attempted to enter them. A fig was deemed as losing receptivity if three successive wasps were not willing to enter it. A total of 458 female figs and 502 male figs were investigated in this way. Temperatures were recorded at the nearby Xishuangbanna Forest Ecology Station.

### Experimental introductions of multiple pollinators

The onset and duration of receptivity on the same male and female trees was detected as before. Pollinator fig wasps were attracted to the bagged receptive figs each morning. We collected groups of ten of these wasps into nylon bags (15×20 cm) that were positioned tightly around figs that had been receptive but un-pollinated for varying lengths of time (twenty figs and 200 female pollinators on each tree each day). This continued until the figs were no longer receptive, which varied between seasons (providing five daily age groups in October 2010, 14 age groups in January 2011 and seven age groups in May 2011).

No fig wasps were still alive on the outside of the figs after one day. We then removed the figs and recorded the locations of the wasps as being either on the outside of the figs, in the ostiole or in the fig cavity. They were recorded as being trapped in the ostiole only if they were facing towards the centre of the figs. Wasps with their heads located in the innermost portion of the ostiole, with their bodies in the cavity, were assumed to have successfully reached the cavity. We measured the head width (the distance between the bases of the eyes) of each individual using an eyepiece graticule mounted on a binocular microscope. The width of the head provides a good indication of body size in this species [11]. Totals of 1040 figs and 10400 pollinators were sampled across the three seasons.

### Experimental introductions of single pollinators

We selected the same two trees as before, and used the same methods for prevention of unwanted pollination events and for detecting the onset of receptivity. We collected mature male figs without exit holes from other trees and stored them in nylon bags. Pollinators were allowed to emerge naturally from the figs and we then gave single recently-emerged individuals the opportunity to enter into male and female figs of known age (20 figs of each age group). We put the pollinators on the surface of the figs at a set distance from the ostioles (15 mm) and recorded two aspects of their behaviour: the time spent walking on the fig surface before contacting the ostiole and the time required to gain entry (taken as the time from when the head was inserted into the first bract until the end of abdomen was inside). We then replaced the bags for the duration of seed and offspring development, which takes several weeks. When the figs were mature, we counted the numbers of seeds in female figs, and pollinator offspring (female and male fig wasps) and empty galls (‘bladders’) in male figs. The numbers of replicates were the same as in the multiple pollinator experiment.

Female head width provides a measure of the quality of the fig wasp offspring produced in figs entered by foundresses at different ages (egg loads in *C. s. marchali* are positively correlated with head width: C. Liu, unpublished). Ten female fig wasp offspring from each of five figs in each age group were initially stored in 70% ethanol with added glycerine. We then measured their head widths as before (a total of 2500 wasps, with sample sizes of 50 wasps for each fig age group).

### Data analysis

We used linear models (LMs) with a priori contrasts to compare the lengths of receptive periods in the three seasons and the body sizes of wasps outside the figs, inside the ostioles and in the fig cavities. We used Wilcoxon’s rank-sum tests to compare the length of receptivity between female and male figs protected from pollinator entry.

We used analysis of variance (ANOVA) to analyze the differences in body size of the pollinators arriving at the male and female trees between seasons and the body size of the wasps between different fig age groups in the multiple pollinators introduction experiment. We also used ANOVA to analyse the body size of offspring on the same days when the foundress entered between different seasons.

We used general linear models (GLMs) to analyze the effects of fig age on biological variables and pollinator behavior (seed and wasp production, the number of empty galls in pollinated figs, sex ratios, the time spent searching for and entering the ostiole, and the final locations of foundresses). Binomial error structures were applied where appropriate (sex ratios, proportions of foundresses in different situations), with Poisson errors applied for count data. We used linear models (LMs) to analyze the relationship between fig age and body size of females in the cavity, trapped in ostiole and outside.

We computed all analyses using R version 2.11.0 (R Development Core Team 2010, Vienna, Austria.)

## Results

### Floral longevity in the absence of pollination

**Figure 1:**
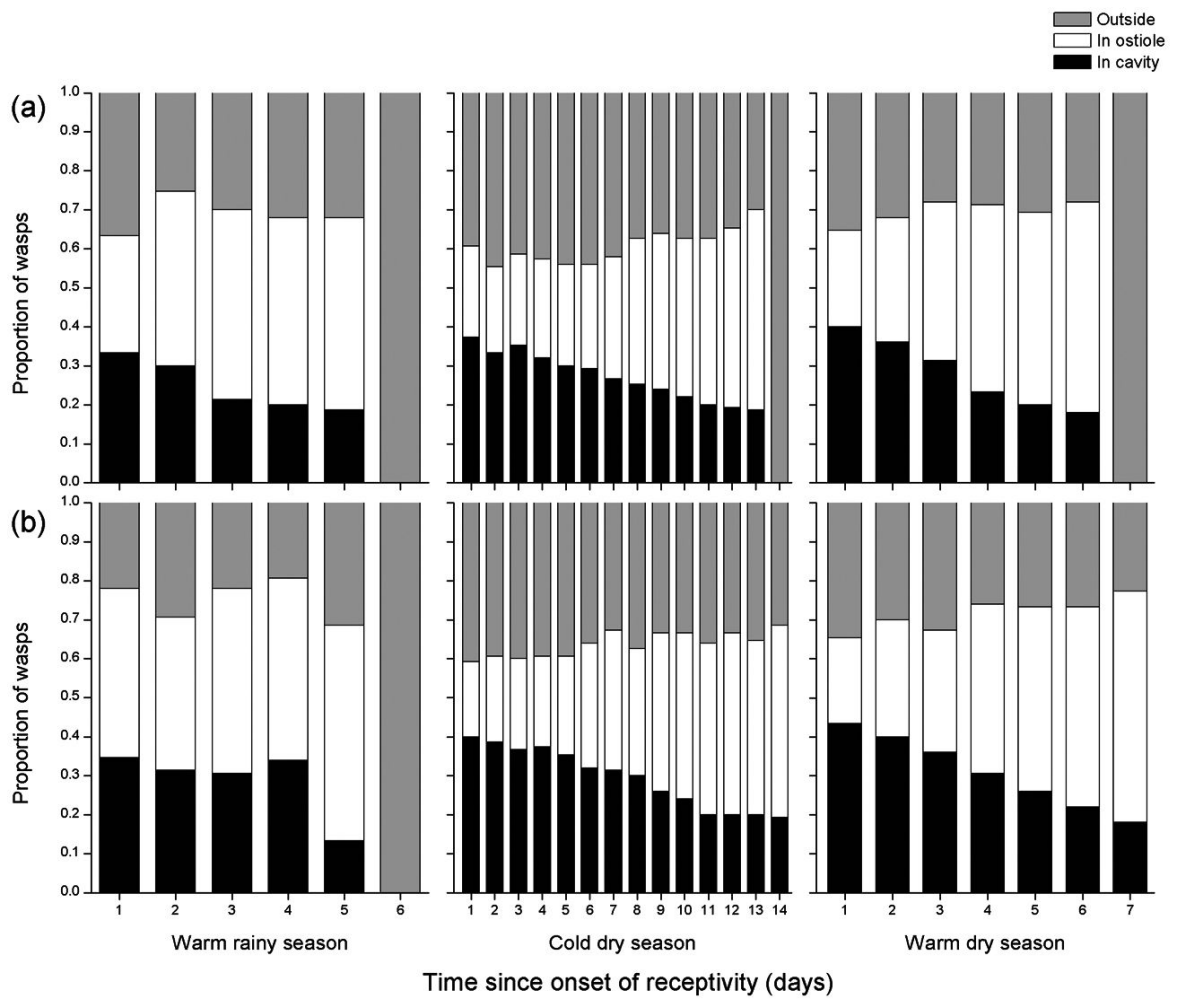
Changes in the willingness and ability of pollinators to enter male and female figs of *F* *. hispida* that had been receptive for different lengths of time. The fig wasps either remained outside the figs, entered but became trapped in the ostiole or succeeded in entering the fig cavity, where they could lay their eggs. Day 1 indicates the first day in receptivity. The experiment was repeated in three seasons. (a) female tree (b) male tree. Note compression of the X axis for the cold dry season in this and subsequent figures.

Figs of *F* *. hispida* that had been protected from pollinator entry remained receptive for 5–7 d during the warm rainy and warm dry seasons, but for up to 14 d in the cool dry season (Figure 1). These differences reflecting seasonal differences in temperatures during the periods of receptivity: 24.30 ± 0.19°C (Mean ± S.E.) in October, 18.76 ± 0.15°C in January, 26.13 ± 0.42°C in May, respectively. Seasonal differences in longevity were similar in male and female figs (Figure 1). Receptivity of male and female figs was of equally short duration in the warm rainy season (Wilcoxon test: W = 150, *P* = 0.099), but male figs remained receptive for slightly longer than female figs in the cold dry season (Wilcoxon test: W = 28, *P* < 0.001) and warm dry season (Wilcoxon test: W = 26, *P* < 0.001)

### The ostiole as a filter of pollinator entry

Not all individuals among the groups of ten pollinators attempted to enter the figs, irrespective of their age (Figure 1) and all fig age groups also had some wasps that had become trapped in their ostioles when they attempted entry. Differences in the proportions of the wasps that attempted entry, became trapped in the ostiole or successfully reached the fig cavity varied consistently with fig age, irrespective of season or fig sex (Figure 1, Table 1). The length of time that figs had been waiting did not influence significantly the proportion of wasps that attempted entry, but the proportion of the wasps trapped in the ostioles significantly increased and the proportion of the wasps reaching the cavity significantly decreased with fig age (Table 1).

**Table 1:** The influence of fig age on the proportions of single pollinator females placed on the surface of figs that either did not attempt entry, became trapped in the ostioles while attempting entry, or succeeded in reaching the cavity in the centre of the figs, where they could oviposit (GLMs with binomial errors).

Category	Fig tree sex	Season	β ± SE	Z	P
Did not attempt to enter	Female	WRS	-0.008 ± 0.002	-0.16	= 0.87
		CDS	-0.02 ± 0.001	-0.73	= 0.52
		WDS	-0.04 ± 0.004	-0.93	= 0.36
	Male	WRS	-0.03 ± 0.005	-0.60	= 0.55
		CDS	-0.02 ± 0.001	-1.63	= 0.10
		WDS	-0.06 ± 0.003	-1.86	= 0.06
Trapped in ostiole	Female	WRS	0.098 ± 0.005	2.06	< 0.01
		CDS	0.07 ± 0.001	6.05	< 0.001
		WDS	0.15 ± 0.03	3.95	< 0.001
	Male	WRS	0.07 ± 0.004	2.94	< 0.05
		CDS	0.07 ± 0.001	6.57	< 0.001
		WDS	0.15 ± 0.003	5.21	< 0.001
Reached cavity	Female	WRS	-0.16 ± 0.006	-2.73	< 0.01
		CDS	-0.06 ± 0.001	-4.45	< 0.001
		WDS	-0.17 ± 0.004,	-4.02	< 0.001
	Male	WRS	-0.14 ± 0.006	-2.57	<0.05
		CDS	-0.06 ± 0.001,	-5.40	< 0.001
		WDS	-0.14 ± 0.003	-4.43	< 0.001

The experiments were replicated in three seasons (WRS = warm rainy season, CDS = cold dry season, WDS = warm dry season).

**Figure 2:**
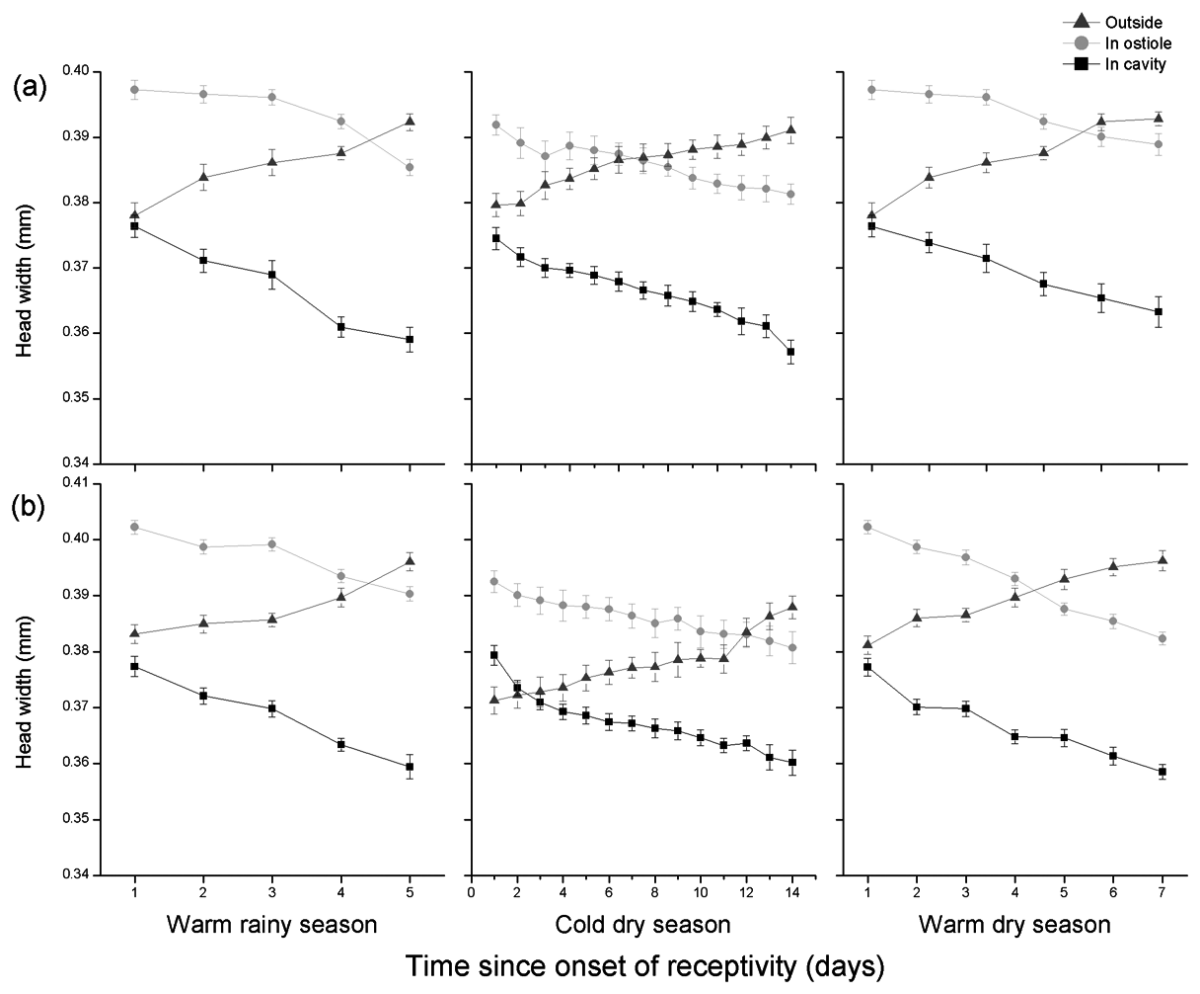
The head widths of pollinator females that remained outside figs of *F* *. hispida* of different ages, became trapped in the ostioles, or succeeded in entering the fig cavities. Day 1 indicates the first day in receptivity. (a) female tree (b) male tree.

**Figure 3:**
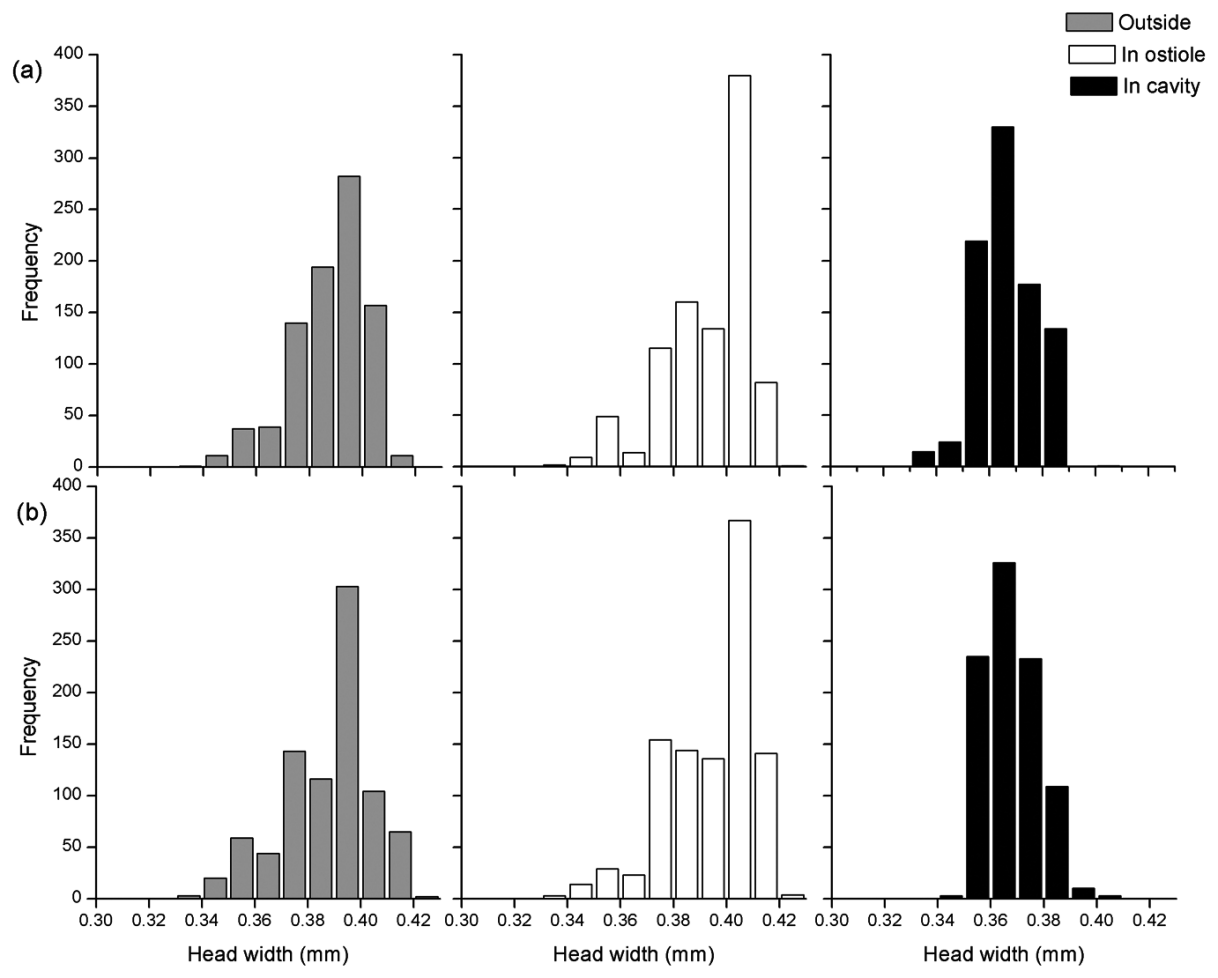
The head widths of pollinator females that remained outside figs of *F* *. hispida* , became trapped in the ostioles, or succeeded in entering the fig cavities (all fig age groups combined). (a) female tree (b) male tree.

The head widths of the pollinators arriving at the male and female trees did not differ between seasons (ANOVA, female tree: *F* = 0.28, *P* = 0.60; male tree: *F* = 0.35, *P* = 0.55). The head widths of the groups of ten fig wasps placed in bags around the figs also did not differ significantly between fig age groups within seasons (ANOVA, female tree: *F* = 1.93, *P* = 0.17; *F* = 0.54, *P* = 0.46; *F* = 0.07, *P* = 0.79 in the three seasons; male tree: *F* = 0.84, *P* = 0.36; *F* = 1.41, *P* = 0.24; *F* = 1.84, *P* = 0.18). The head widths of individuals that failed to attempt entry significantly increased as the figs got older, but the wasps trapped in the ostiole and those that reached the cavity became significantly smaller – larger individuals preferred to attempt entry into younger figs (Figure 2, Table 2). This resulted in a decline in the mean head widths of those wasps that had attempted to enter the figs. Head widths among wasps that had become trapped in the ostiole and wasps that succeeded in reaching the fig cavity declined in parallel with fig age, with wasps that had become trapped always larger (Figure 2). Among the wasps that attempted entry, the proportion that was successful therefore remained broadly unchanged. Consequently, in young figs the wasps that remained on the outside of the figs were about the same size as those that reached the fig cavity, but in the oldest figs there was a large difference in size because the larger individuals were less likely to enter. These age-related changes in body sizes were consistent between seasons and between male and female figs (Figure 2). The overall effect of differences in willingness to enter and success in passing through the ostiole resulted in the wasps remaining outside the figs being similar in size to those trapped in the ostiole, but with wasps that reached the fig cavity significantly smaller (Figure 3) （Outside vs. in cavity: LM: β ± SE = 0.014 ± 0.0008, t = 17.08, *P* < 0.001; trapped in ostiole vs. in cavity: LM: β ± SE = 0.015 ± 0.0007, t = 21.94, *P* < 0.001). Very few females with head widths greater than 0.39 mm managed to successfully negotiate the ostiole.

**Table 2:** The sizes of pollinator females that when placed singly on the surface of figs either failed to enter, entered but became trapped in the ostiole or successfully reached the fig cavities, in relation to the ages of the figs (LMs for head width).

Category	Fig tree sex	Season	β ± SE	t	P
Did not attempt to enter	Female	WRS	0.003 ± 0.0004	7.40	< 0.001
		CDS	0.001 ± 0.0003	3.28	< 0.001
		WDS	0.003 ± 0.0003	9.08	< 0.001
	Male	WRS	0.002 ± 0.0005,	4.34	< 0.001
		CDS	0.001 ± 0.0002	5.25	< 0.001
		WDS	0.002 ± 0.0004	6.50	< 0.001
Trapped in ostiole	Female	WRS	-0.003 ± 0.001	-5.62	< 0.001
		CDS	-0.001 ± 0.0002	-3.07	< 0.001
		WDS	-0.002± 0.0004	-5.15	< 0.001
	Male	WRS	-0.003± 0.0004	-6.37	< 0.001
		CDS	-0.001 ± 0.0002	-4.15	< 0.001
		WDS	-0.03 ± 0.0003	-10.62	< 0.001
Reached cavity	Female	WRS	-0.005 ± 0.001	-7.32	< 0.001
		CDS	-0.001 ± 0.0001	-7.14	< 0.001
		WDS	-0.002 ± 0.001	-5.22	< 0.001
	Male	WRS	-0.004 ± 0.001	-8.46	< 0.001
		CDS	-0.001 ± 0.0001	-8.95	< 0.001
		WDS	-0.03 ± 0.0003	-7.63	< 0.001

The experiments were replicated in three seasons (WRS = warm rainy season, CDS = cold dry season, WDS = warm dry season).

**Figure 4:**
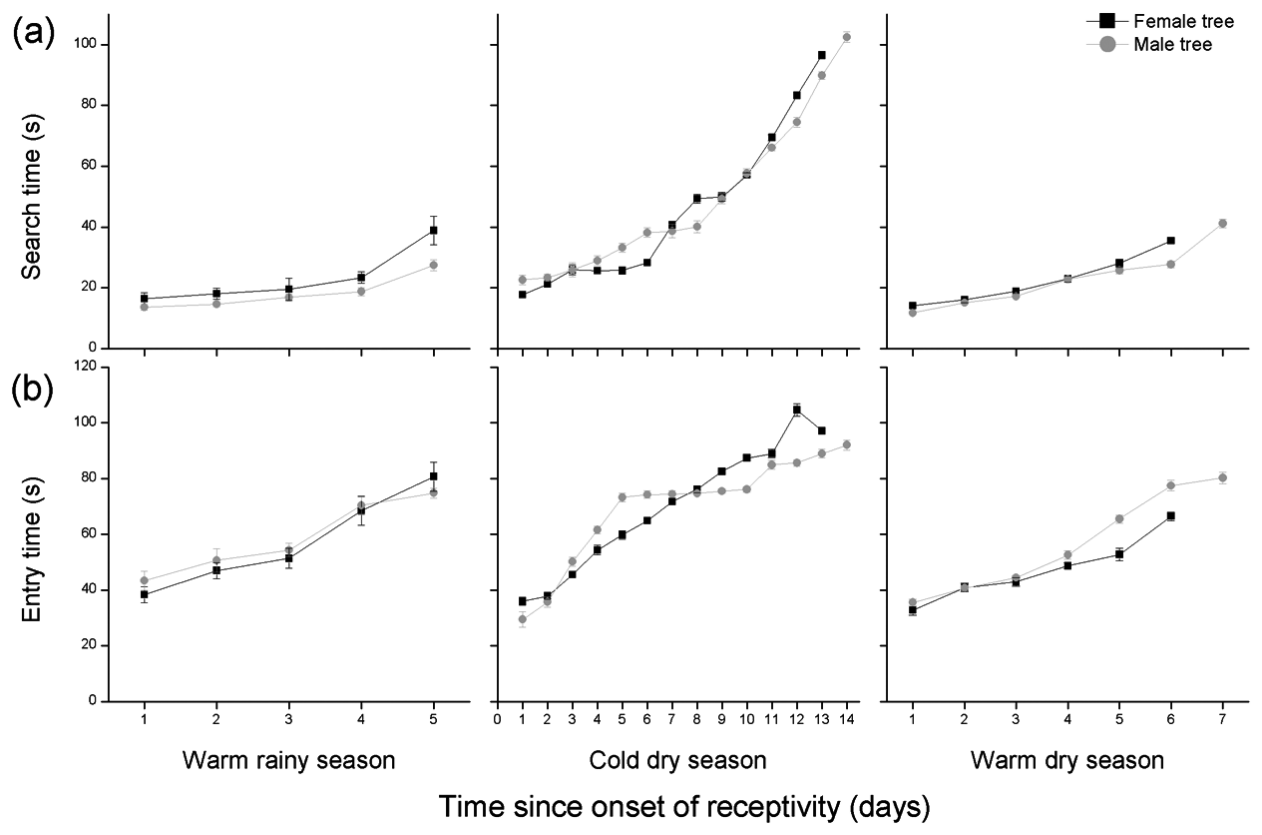
The behaviour of pollinators when placed on the surface of figs of different ages at a fixed distance from their ostioles (a) the time taken by female pollinators to reach the ostioles and (b) the time required by the wasps to insert fully their bodies into the ostioles, recorded from when they first inserted their heads. Day 1 indicates the first day of receptivity.

When single females were placed on the surface of figs, the time they spent looking for the ostiole increased with fig age, suggesting that older figs were less attractive (Figure 4, Table 3). Among those that decided to then enter the ostioles, it also took longer for them to penetrate into older figs. These results were again consistent across sexes and seasons, though the search times and entry times were generally longer in the cold dry season, when temperatures were lower.

**Table 3:** The times taken by single pollinator females placed on the outside of figs in relation to the ages of the figs and the times required to fully enter the ostioles (GLMs with Poisson errors).

Category	Fig tree sex	Season	β ± SE	Z	P
Time before deciding to enter	Female	WRS	0.22 ± 0.02	14.57	< 0.001
		CDS	0.14 ± 0.003	53.06	< 0.001
		WDS	0.19 ± 0.01	16.36	< 0.001
	Male	WRS	0.18 ± 0.02	10.51	< 0.001
		CDS	0.12 ± 0.003	47.65	< 0.001
		WDS	0.19 ± 0.009	21.15	< 0.001
Time to fully enter the ostiole	Female	WRS	0.19 ± 0.01	19.70	< 0.001
		CDS	0.08 ± 0.002	40.48	< 0.001
		WDS	0.13 ± 0.01	16.19	< 0.001
	Male	WRS	0.13 ± 0.01	13.65	< 0.001
		CDS	0.03 ± 0.002	14.01	< 0.001
		WDS	0.07 ± 0.006	11.16	< 0.001

The experiments were replicated in three seasons (WRS = warm rainy season, CDS = cold dry season, WDS = warm dry season).

### Age-related changes in fig productivity

**Figure 5:**
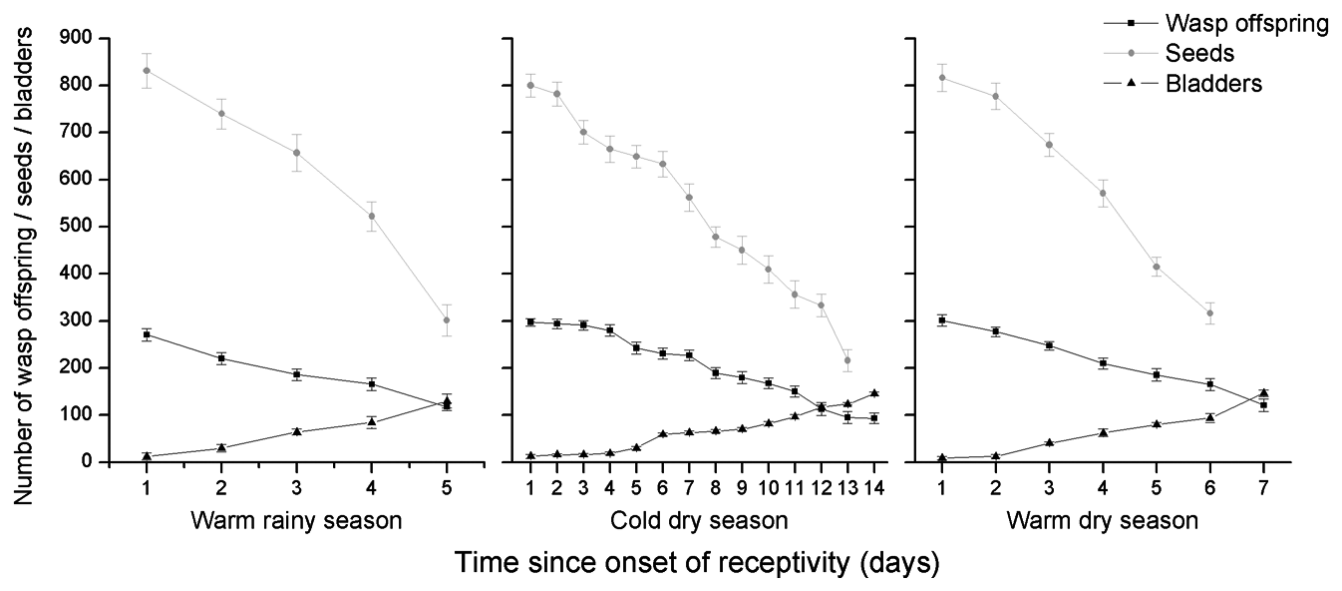
The contents of *F* *. hispida* figs that had been entered by single pollinators at different ages. The numbers of seeds were recorded in female figs, wasp offspring and empty galls in male figs. Day 1 indicates the first day in receptivity.

Figs where a single female fig wasp had been allowed entry produced the highest number of seeds and wasps on the first day the figs were receptive, with productivity then declining significantly with fig age (Figure 5, Table 4). Although productivity in both female and male figs declined with age, the declines in seed production were much more marked than the declines in wasp offspring. Despite this, there were consistently more seeds in female figs of all age groups than there were pollinator offspring in male figs of the same age (Figure 5). In male figs the significant decline in fig wasp offspring production was associated with a fig age-related increase in the numbers of empty galls, implying that much of the decline in pollinator offspring production was attributable to higher failure rates among galled ovules. The females that succeeded in reaching the fig cavity of older figs were also smaller on average than those that could enter and oviposit in younger figs. Because egg loads in *C. s. marchali* are positively correlated with head width (C. Liu, unpublished), the smaller entrants into older figs will also have had fewer eggs to lay.

**Table 4:** Variation in seed, empty gall and pollinator offspring numbers in relation to the age of figs at the time of entry by a single pollinator (GLMs with Poisson errors).

Category	Fig tree sex	Season	β ± SE	Z	P
Seeds	Female	WRS	-0.10 ± 0.003	-32.87	< 0.001
		CDS	-0.08 ± 0.001	-99.43	< 0.001
		WDS	-0.16 ± 0.002	-59.11	< 0.001
Fig wasp offspring	Male	WRS	-0.19 ± 0.006	-32.43	< 0.001
		CDS	-0.08 ± 0.001-	-61.74	< 0.001
		WDS	-0.13 ± 0.003	-40.84	< 0.001
Empty galls	Male	WRS	0.56 ± 0.001	47.67	< 0.001
		CDS	0.14 ± 0.002	58.67	< 0.001
		WDS	0.29 ± 0.007	42.92	< 0.001

The experiments were replicated in three seasons (WRS = warm rainy season, CDS = cold dry season, WDS = warm dry season).

**Figure 6:**
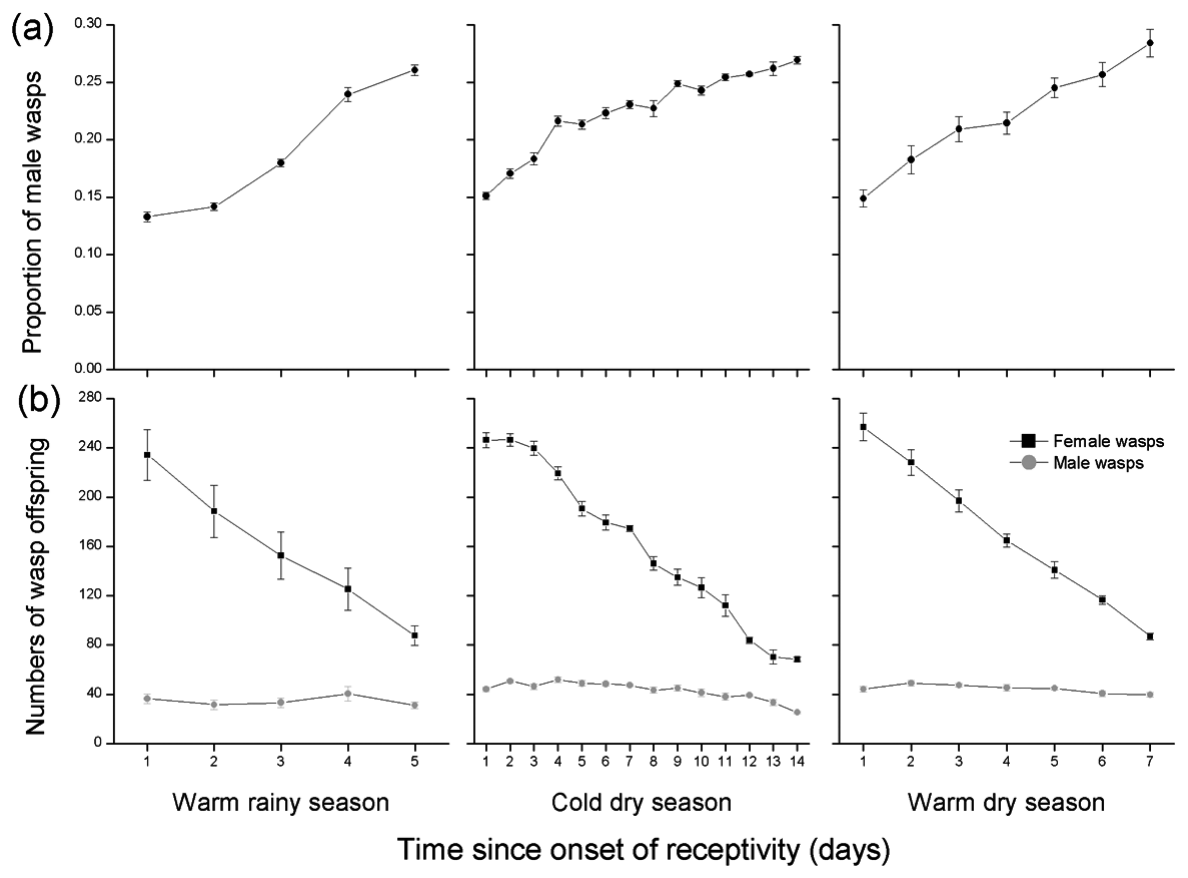
Changes in the numbers and sex-ratios of pollinator offspring in male *F* *. hispida* figs entered at different ages (a) Sex ratio (proportion of male offspring) (b) The numbers of female and male offspring. Day 1 indicates the first day in receptivity.

**Figure 7:**
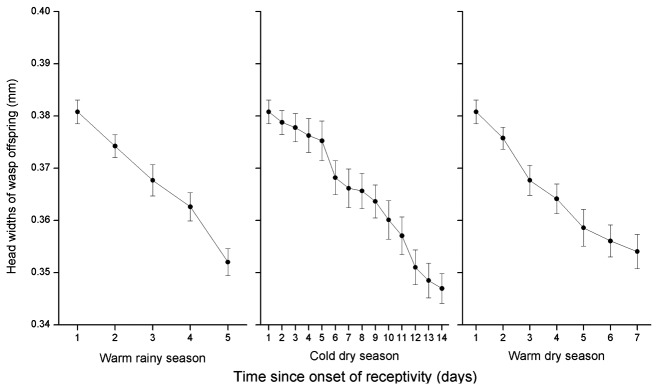
The head widths of female pollinator offspring from male *F* *. hispida* figs entered at different ages. Day 1 indicates the first day in receptivity.

The decline in offspring numbers with fig age among figs entered by a single foundress was caused by a significant decline in the numbers of females but not males, and resulted in significant age-related changes in offspring sex ratios, from about 0.15 in young figs to more than 0.25 in the oldest figs (Figure 6, Table 5). The female offspring emerging from older figs were also smaller (Figure 7, Table 5). The offspring of foundresses that entered figs on the first day of receptivity were of similar size in all three seasons (ANOVA, *F* = 0.03, *P* = 0.87), but the slower rate of change among figs in the cool dry season meant that this was not the case for figs that had been waiting five days, with offspring produced in that season significantly larger than wasps in day 5 figs during the other two seasons (LM, cold dry season vs. warm rainy season: β ± SE = 0.01 ± 0.003, t = 3.91, *P* < 0.001; cold dry season vs. warm dry season: β ± SE = 0.02 ± 0.003, t = 5.25, *P* < 0.001). Overall, wasp offspring emerging from figs in the cool dry season were no smaller than those in the other seasons (ANOVA, *F* = 0.96, *P* = 0.33).

**Table 5:** Variation in the numbers, sexes and female head widths of offspring resulting from the entry of single females into figs of varying ages (GLMs with Poisson errors for changes in the numbers of male and female offspring, GLMs with binomial errors for offspring sex ratios and LMs of female offspring head widths).

Category	Season	β ± SE	Z	P
Female offspring	WRS	-0.23 ± 0.06	-35.5	< 0.001
	CDS	-0.09 ± 0.001	-61.99	< 0.001
	WDS	-0.17 ± 0.004	-44.17	< 0.001
Male offspring	WRS	-0.001 ± 0.01	-0.11,	0.912
	CDS	-0.007 ± 0.01	-0.78,	0.328
	WDS	-0.005 ± 0.003	-0.66,	0.517
Proportion male	WRS	0.19 ± 0.01	12.81	< 0.001
	CDS	0.04 ± 0.003	13.97	< 0.001
	WDS	0.11 ± 0.008	13.29	< 0.001
Female offspring head width	WRS	-0.007 ± 0.001	-8.58	< 0.001
	CDS	-0.003 ± 0.0002	-12.85	< 0.001
	WDS	-0.005 ± 0.001	-8.46	< 0.001

The experiments were replicated in three seasons (WRS = warm rainy season, CDS = cold dry season, WDS = warm dry season).

## Discussion

The ostiole of *F* *. hispida* represents a significant challenge for foundress female fig wasps attempting to reach their oviposition sites inside the figs. Under natural conditions, more than half the *C. s. marchali* females that attempt to enter the figs of *F* *. hispida* at XTBG become trapped in the ostioles [37] and at least one in three females in our experiments became trapped before they had a chance to reproduce. There is also a strong advantage for single wasps entering figs that have only recently become receptive – the wasps are less likely to become trapped. The wasps also took longer to force their way through the outer ostiolar bracts of older figs, suggesting that over time the bracts that form the ostiole became less flexible and more difficult for the wasps to displace. Ostiole penetration is especially problematic for larger females, because they are more likely to become trapped than smaller ones. In apparent response, larger females are more reluctant to enter older figs. The filtering effects of the ostiole are reflected in the next generation of fig wasps, with fewer and smaller offspring present in older figs. This need not have a genetic basis as older figs may also have fewer flowers that remain suitable for oviposition and also (based on the increase in numbers of failed, empty galls) appear to provide poorer quality resources for the wasps’ larvae. From the plant’s perspective, older figs on male trees are less productive than younger ones, producing fewer female pollinators to transport their pollen, with older figs on female trees (where the wasps can never reproduce) also producing fewer seeds.

Most adult female fig wasps probably survive for just a single flight period of a few hours. After what may have been a long flight, this gives them little time to select which fig to enter from among the figs that are available. Host choice is particularly acute for pollinators of dioecious fig trees, because entry into female figs always means that they will fail to reproduce. Pollinators nonetheless fail to discriminate between male and female figs, a failure that has been attributed to inter-sexual mimicry [38] and their overwhelming need to enter figs as quickly as possible: ‘selection to rush’ [31]. Our results show that pollinator females are more discriminating than previously realized: they prefer younger figs and larger individuals are less likely to attempt entry than smaller ones. Given the lower productivity of older figs, the greater difficulty in entering older figs and the lower likelihood that a larger female will succeed, these behavioural differences can all be interpreted in terms of adaptations that improve the survivorship and reproduction of the wasps. Smaller wasps are less discerning than larger individuals, but can afford to be as they are also more likely to successfully gain entry into the figs. They may need to decide on entry more quickly because smaller individuals are likely to be more prone to dehydration.

The extent to which adult body size is a heritable trait in fig wasps is unknown, but environmental influences are typically strong in insects [39]. Adult fig wasps do not feed, so body size and fecundity are determined before eclosion [11]. Rates of larval development are strongly influenced by temperature and shorter development times will often be advantageous [40]. Along with the greater risks of parasitism associated with a prolonged development period, there is also intense scramble competition for mates among pollinator males that will favour individuals that can complete their development more quickly and emerge first. Fig wasp body size is likely to be influenced by the quality of the nutrition provided by their host figs, which may vary between figs and between trees. Body size is also influenced by the location of larval development within individual figs – adult female *C. s. marchali* that emerge from galls nearer to the central cavity of *F* *. hispida* figs are larger (Y.-Q. Peng unpublished).

The highly localized mating behaviour of pollinator fig wasps has made them a popular group for studies of sex ratio evolution [27,41,42]. Comparisons between theoretical optima and observed changes in fig wasp offspring sex ratios with the numbers of foundresses entering a fig generally show good qualitative agreement, but usually also considerable unexplained variation. Less female-biased offspring sex ratios in figs with multiple foundresses are often generated by a ‘mostly male eggs first’ oviposition strategy in combination with increased competition for oviposition sites that reduces the average clutch size of each female [27]. At least some *Ceratosolen* species have this oviposition sequence where less female-biased offspring sex ratios are expected to result whenever individual clutch sizes are reduced (H. Wang, unpublished). Consistent with this, individual *C. s. marchali* females that entered older figs had smaller numbers of female offspring and age-related variation in host quality was therefore responsible for a change in fig wasp sex ratio. However, older figs also contained more empty galls, each of which is likely to have initially contained a fig wasp egg, so a higher survival rate among male offspring may also have contributed to this relationship [43]. The two mechanisms may be complementary, because earlier-generated galls (containing males) may compete better for limited resources within the figs [44].

The long period of floral receptivity in *F* *. hispida* is typical for *Ficus* species [20,21,36]. It increases the probability that un-pollinated figs will overlap with the arrival of pollinators released from figs on other trees, but is achieved at the cost of reduced productivity among older figs on both male and female trees. Similar results have been described in other *Ficus* species [21,26]. Ostioles are usually described as opening at the beginning of the receptive phase and closing rapidly at the end, once pollinators have entered [17,30,45,46]. Our results suggest that ostiole closure in un-pollinated figs is an incremental and slower change associated with a decline in attractant volatiles released by the figs and increasing difficulties for pollinators that try to enter.

Floral longevity in *F* *. hispida* at XTBG is much longer during the cold dry season – a widespread plant response to cooler temperatures. Other phases of fig development in *F* *. hispida* are also extended during the cold dry season, so daily releases of pollinators from mature figs are also much reduced. Figs entered during the colder season will therefore be older on average than figs pollinated during warmer seasons, but their slower rate of decline with age can compensate, so cold season figs will not necessarily produce fewer seeds or pollinators.

The high frequency of trapped pollinators in *F* *. hispida* figs appears to be detrimental to the plant. It reduces or entirely prevents pollen entry into female figs and reduces the numbers of pollinator offspring that could develop in male figs. The ostiole of *F* *. hispida* also favours smaller pollinators, which are less successful at dispersing between trees, and less fecund. Ostiolar structure varies greatly between *Ficus* species [12,35], and even if the ostiole of *F* *. hispida* proves to be unusually difficult to penetrate, there is clearly a reproductive cost to the tight ostiole that is so characteristic of the genus.

This study highlights costs to fig trees associated with their extended floral longevity and also how fig wasp pollinators respond to declines in resource quality associated with fig aging by being more likely to enter younger figs, where their own reproductive success will be enhanced. Furthermore, responses to the aging of their oviposition sites are not uniform, with larger pollinators more responsive to fig age than smaller individuals. Figs clearly vary in the quality of the resources they offer to their pollinators and their host choices reflect this, despite the constraints imposed on them by their short adult life spans. The particular reluctance of larger pollinators to enter older figs can be explained by the greater likelihood that they will become trapped, but their responses may also be influenced by physiological features linked to their greater body size – larger individuals may be dehydrating more slowly for example. The consequences of these interactions suggest that whereas the ostiole of *F* *. hispida* sets an upper limit to the body size of its pollinator, there is no simple optimal body size for *C. s. marchali* , because this will vary according to variables that include the age of the figs that it encounters when seeking to lays its eggs. 
